# Nutritional Regulation of Mammary Gland Development and Milk Synthesis in Animal Models and Dairy Species

**DOI:** 10.3390/genes12040523

**Published:** 2021-04-03

**Authors:** Cathy Hue-Beauvais, Yannick Faulconnier, Madia Charlier, Christine Leroux

**Affiliations:** 1 INRAE, AgroParisTech, GABI, University of Paris-Saclay, F-78350 Jouy-en-Josas, France; madia.charlier@inrae.fr; 2 INRAE, VetAgro Sup, UMR Herbivores, University of Clermont Auvergne, F-63122 Saint-Genès-Champanelle, France; yannick.faulconnier@inrae.fr (Y.F.); christine.leroux@inrae.fr (C.L.)

**Keywords:** mammary gland, nutrition, lactation, milk, development, gene expression

## Abstract

In mammals, milk is essential for the growth, development, and health. Milk quantity and quality are dependent on mammary development, strongly influenced by nutrition. This review provides an overview of the data on nutritional regulations of mammary development and gene expression involved in milk component synthesis. Mammary development is described related to rodents, rabbits, and pigs, common models in mammary biology. Molecular mechanisms of the nutritional regulation of milk synthesis are reported in ruminants regarding the importance of ruminant milk in human health. The effects of dietary quantitative and qualitative alterations are described considering the dietary composition and in regard to the periods of nutritional susceptibly. During lactation, the effects of lipid supplementation and feed restriction or deprivation are discussed regarding gene expression involved in milk biosynthesis, in ruminants. Moreover, nutrigenomic studies underline the role of the mammary structure and the potential influence of microRNAs. Knowledge from three lactating and three dairy livestock species contribute to understanding the variety of phenotypes reported in this review and highlight (1) the importance of critical physiological stages, such as puberty gestation and early lactation and (2) the relative importance of the various nutrients besides the total energetic value and their interaction.

## 1. Introduction

In mammals, milk must provide neonates not only essential nutrients but also a complex repertoire of agents necessary for healthy development. The consumption of milk also supports the growth of children and accompanies adults throughout their lives. The nutritional value of milk for humans has led the scientific community to increase the health benefits of milk nutritional qualities in the context of the evolution of farming, ensuring its added value, economic performance for breeders, and animal welfare. Milk is secreted by the mammary gland (MG), and the development and function of this organ influence the efficiency of milk production and composition. Understanding the process of MG development is crucial for designing effective levers for improving milk production and composition [1]. Mammary gland development takes place from the fetal stage and then continues during critical periods of life, which are peri puberty, pregnancy, and lactation [2]. The cyclicality of mammary function, driven by the reproductive cycle, makes the MG an organ that entices scientists.

Milk is composed of significant components, including proteins, lipids, and oligosaccharides mainly lactose, vitamins, nucleic acids, and cells. Milk composition depends on species and offspring needs [3] and sex [4]. Nutrition has considerable effects on MG development and on milk composition and these effects may be reversible and rapid. The impact of nutrition on MG development and lactation has been studied extensively. Animal models are relevant for investigating nutritional regulation of MG development and subsequent lactation as well as underlying mechanisms [5,6]. After the nursing period, the human consumption of milk involves ruminant milk. Thus, in recent decades, the nutritional value of milk, defined by its composition, has attracted the attention of producers and consumers and aroused the curiosity of scientists, focusing on improvement of milk nutritional quality. In particular, the milk lipid fraction has been studied extensively due to its fatty acid (FA) composition and the various effects of FAs on human health [7,8]. In ruminants, the rumen plays an important role in milk FA composition. The dietary lipids of ruminants are almost completely hydrolyzed, and unsaturated FAs are mainly hydrogenated in the rumen, whereas in monogastrics, dietary FAs arrive at the intestine without modifications [9]. Due to this species specificity and the crucial role of FA composition in human health, the mechanism of regulation of milk component biosynthesis and secretion has been deciphered in ruminants.

**Figure 1:**
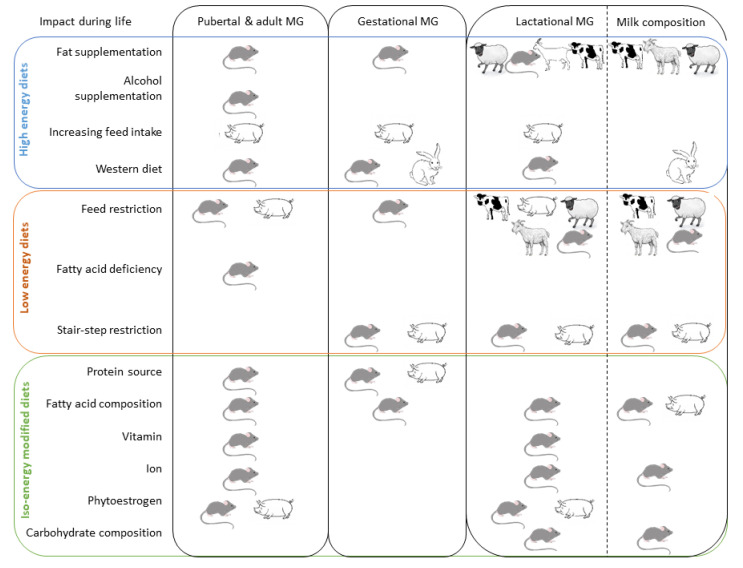
Summary of the discussion of the impact of nutrition on mammary development and milk composition, according to species, physiological stages, and type of diet used.

The objective of this article is to providean overview of the effects of nutrition on MG from its development to regulation of milk synthesis. In this paper, we review the available knowledge and understanding of the nutritional regulation of MG development and lactation, on the one hand in three animal species (rodents, rabbits and pigs), considering the nutritional regulation of the MG development, and on the other hand, in three ruminant species, regarding the nutritional regulation of gene expression involved in the synthesis of milk components. (Figure 1). Mammary gland development strongly influences milk quantity and quality. Indeed, mammary epithelial cells proliferation directly impacts milk production, their differentiation leads to the synthesis and secretion of specific components in milk. Moreover, the importance of the nutritional quality of milk for human health and the economic and social value of milk consumption led us to consider the molecular mechanisms regulating the biosynthesis and secretion of milk components in ruminant species. Data concerning the influence of nutrition on mammary tumorigenesis or susceptibility to cancer incidence are not included in this review, nor are in vitro studies carried out on mammary cells.

## 2. Nutritional Regulation of the Mammary Gland Development

During puberty, the MG undergoes development, which is characterized by the onset of large bulbous terminal end buds, leading to the infiltration of branching epithelial ducts through the mammary tissue [10]. During pregnancy, the ductal epithelium of the mammary anlage invades the mammary fat pad, and this phenomenon is accompanied by strong epithelial cell proliferation and differentiation, which will lead to successful lactation. These processes are tightly regulated by hormones such as sex steroids, prolactin, and growth hormone, the main drivers of MG development [11]. Adequate nutrition is of upmost importance during these periods of rapid and profound physiological changes in mammary tissue. Three animal models (rodents, rabbits, and pigs) have been mainly used for studying impacts of nutritional challenges from early life and during every developmental stage of the MG.

## 3. Effects of Energetic Supplementation

Food energy is defined as the energy released from carbohydrates, fats, proteins, and other organic compounds. Energy released by a particular food is a critical parameter in nutrition. Hyperenergetic feeding can result either from a quantitative increase in the ration or from a qualitative modification of the ration, thus increasing the energy intake while maintaining an identical quantity of food by modifying the carbohydrate, protein, or fat content. The main types of hyperenergetic diets used are high-fat [12,13] and/or high-carbohydrate [14,15] diets, often associated with a decrease in the protein rate of the ration, or even a Western diet rich in fat and sugar and often salt, combined with a deficiency in vitamins and minerals that are essential for good health [16,17]. Although an increase in animal energy intake is associated with diseases such as cardiovascular diseases, diabetes, and cancer risk [18], its effect on mammary development has not yet been fully documented, particularly regarding the importance of different physiological stages.

*Ad libitum*-feeding during the prepubertal period in pigs exhibits increased energy intake and enhanced mammary development [19], by promoting mammary epithelial development, including an increase in parenchymal tissue [20,21]. Similarly, additional studies have shown that mammary growth is affected by nutrient intake during lactation. Increasing feeding allows the provision of adequate amounts of nutrients, which can be directly correlated with increased mammary growth and milk yield as well as litter weight gain [22].

On the contrary, increased adiposity caused by high-fat diets, specifically during puberty, delays MG development, characterized by stunted mammary duct elongation and reduced mammary epithelial cell proliferation [23]. Structural analyses of the MGs of nulliparous nonpregnant mice fed a high-fat diet revealed an enlarged fat pad size and a less dense distribution of ducts with less frequent branching. Additionally, the ducts were surrounded by thick collagen layers and were incompletely lined with myoepithelial cells. These morphological changes have been correlated with high expression of leptin [24], which has been involved in the control of mammary epithelial cell proliferation [25]. These results suggest that high-fat diet consumption during critical life-time periods, such as early life, impairs mammary ductal development by remodeling the mammary microenvironment and promoting the expression of paracrine regulators. Moreover, MGs from lactating rats fed a high-fat diets displayed a disrupted morphology characterized by a few and small alveoli and were associated with a decrease in the transcription process in basal epithelial cells, which are important to the differentiation of the MG [26]. Although the measurement of milk production is difficult in models such as rodents, a high-fat diet consumed from puberty to mid-lactation showed no significant differences in milk yield during the first half of lactation [27]. Nevertheless, effects of high-fat diet tightly depend on species. Using a rabbit model with females fed a high-fat/high-sugar diet consumed before puberty to pregnancy, MG development was markedly modified, showing enhanced mammary differentiation, affecting milk composition [28,29]. Moreover, feeding rabbits with high fat/high sugar diets during the only peri-pubertal period induces modifications in relative proportion of mammary epithelial tissue at mid-pregnancy, confirming puberty as a critical window of nutritional regulation as equally shown in livestock [30,31]. In rabbits, these morphological modifications are accompanied by a precocious synthesis of major milk proteins and an abnormal secretion of large lipid droplets and numerous micelles in the alveolar and ductal lumen [28]. These data contrasted with findings using mice fed a similar cafeteria-type diet starting at or after puberty, in which impaired mammary development was observed before pregnancy [24], at mid-pregnancy, and during lactation [32], thus suggesting species-specific nutritional regulation of MG development.

Although morphological alterations following energetic supplementation have been extensively described, underlying mechanisms are not yet fully understood. Following puberty, MG development is tightly regulated by the interaction of the mammary epithelium with the stromal compartment. Estrogens regulate cellular function by modulating the expression and activity of genes involved in signal transduction pathways, including the protein kinase C (PKC) family [33,34]. Endocrine and molecular studies have shown that a high-fat diet increases PKC activity and estrogen receptor level in mammary tissue [35,36]. Furthermore, consumption of a high-fat diet alters the transcriptome of mammary tissue at the peak of lactation and creates a proinflammatory environment [27]. A hyperenergetic diet can alter the gene expression profile in the MG during lactation and thus modify the synthesis of milk components and subsequent milk composition. In particular, the expression of a variety of collagen genes was disrupted in the MG of female mice fed a high-fat diet, which is consistent with previous studies in other tissues such as the liver and heart [27,37,38]. After a high-fat diet, mammary tissue exhibits an inflammatory process that can lead to impaired synthesis and secretion of milk. Molecular analyses showed a downregulation in milk protein genes and an increased expression of inflammation-related genes [26], some of which may be involved in mammary duct development [39]. In the range of consequences on metabolism, stearoyl-coA desaturase 1 (*Scd1*), a key enzyme of FA metabolism, is one of the most highly expressed genes in mammary tissue of high-fat diet-fed mice, suggesting a direct correlation between dietary fat and milk monounsaturated FA concentrations and highlighting the relationship between diet and lactation [40]. Recent data have proven that *Scd1* upregulation is driven by epigenetic mechanisms such as reduced methylation in the *Scd1* promoter in adipose tissue of high-fat diet-fed rats [41].

To create models intended to mimic the dietary deregulation found in humans and study its effect on mammary development, diets combining excess fat with excess sugar have been used. A Western diet, also called an obesogenic or cafeteria diet, provides excessive energy intake due to high fat and salt content but is associated with a deficiency in calcium and vitamin D [42]. The effects on MG development observed with Western diets are not identical to those obtained with high-energy diets. Prolonged consumption of Western diet from puberty onwards, revealed structural modifications in the mammary development of adult mice. An increased number of mammary ducts and a higher proliferation of cells located in the small terminal ducts in Western diet-fed mice have been observed [43]. Moreover, Medina et al. reported an increased size of the proliferative epithelial cell compartment and an excessive duplication of mammary ductal epithelial cells [44], consistent with studies, demonstrating that a high-fat diet combined with calcium deficiency, induces hyperproliferation of mammary cells in the terminal structures [45]. These deleterious effects in the MG can be reduced or even reversed by adding dietary calcium and antioxidant mixtures to Western diets [46]. In a similar model, Xue et al. also described hyperplasia due to the hyperproliferation of mammary epithelial cells, putatively reversible by adding calcium and vitamin D3 to the diet, thus suggesting that micronutrient deficiency may be more deleterious than fat excess [47,48]. While the impact of rich lipid diets has been widely studied in ruminants due to the nutritional value of milk for human health, their impact on milk composition in non-ruminant mammals remains poorly understood [49,50,51]. In lactating mice, the cafeteria diet consumption induces a reduction in the whey acidic protein content, concomitant with an increase in serum albumin and lactoferrin contents as well as in the phosphorylated isoforms of the main milk proteins in early and mid-lactation [29].

Increasing dietary protein levels as part of an increase in total energy of the whole diet during early pregnancy in gilts did not benefit mammary development, but the same diet has been shown to be deleterious to mammary epithelial tissue development in late pregnancy [52], highlighting the importance of the stage in the nutritional regulation of the MG development. In contrast, in the same species, elevated dietary energy intake, as well as the addition of soybean oil, seems to have no effect on MG development in late pregnancy [19,53]. Moreover, in a rodent model, energy intake was increased by alcohol addition to the diet. A delay in the maturation of mammary tissue has been observed and was characterized by a decrease in the density of alveolar bud structures and a decrease in serum progesterone, which plays a key role in MG epithelium maturation in young female rats [54]. Taken together these data clearly demonstrate that the consequences of an energetic supplemented diet do not depend exclusively on the caloric excess, but more subtly on the source of this excess.

## 4. Effects of Energetic Restriction

In breeding, energetic restriction is an effective dietary alternative to prevent chronic diseases such as cardiovascular defects, diabetes, and dyslipidemia [55,56]. However, the positive effects of energetic restriction, on an organ such as the MG, which develops throughout life, remain a key issue.

Low-energy diets negatively impact mammary development, as shown in mice, where 40% caloric restriction was associated with a decrease in mammary weight and an atrophied ductal tree, characterized by a less representative area of epithelial tissue and a reduced size of mammary adipocytes. Underfeeding during pregnancy significantly impaired MG development, as observed by the decreased mammary mass and the DNA and protein contents, in pregnant females fed with half-reduced food intake [57]. Another half-restricted intake model was used in rats to study the effect of undernutrition during pregnancy on mammary development. The mammary mass was markedly reduced, as were the DNA, RNA, and protein contents, while the RNA/DNA ratio was conserved. Histological analyses showed diminished fat accumulation in the MG and a lower number of alveolar and ductal epithelial cells. During lactation, the mass of mammary tissue was severely reduced in low-calorie, protein-depleted diet-fed dams, and the protein content was modified in the secretory tissue, possibly inducing a moderating effect on gene regulation [58]. As in rodents, in pigs, when restrictive feeding was applied before puberty [21,59], or from early life throughout puberty until adult age [20,21], impaired mammary parenchymal development was observed. Taken together, these data demonstrate that hypocaloric intake impacts the physiological hyperplasic and hypertrophic response of the secretory tissue and is associated with a diminished quantity of secretory cells, characterized by reduced cytoplasm and synthesis activity [57]. However, an enhancement of the mammary stem cell self-renewal was observed during caloric restriction [60] suggesting that the impact of nutritional restriction depends on the mammary cell lineages.

As for molecular mechanisms involved, the mTOR/Akt signaling pathway, which regulates cell growth, transcription, differentiation, and survival, is strongly modulated by caloric restriction, as reported in nonmammary tissues [61,62,63]. When dietary energy restriction from 0% to 40% was applied in rodents, molecular analyses of mammary intracellular energy sensing pathways showed that the levels of phosphorylated mammalian targets of rapamycin (mTOR) decreased with the percentage of restriction in a dose-dependent manner. In addition, this downregulation of mTOR was correlated with low expression of Akt [64,65].

Experiments involving low-calorie diets have highlighted the importance of FAs such as linoleic acid on cell physiology and organogenesis, particularly during the MG development process. Mice fed a linoleic acid-deficient diet showed a decrease in fat in the mammary fat pad and impaired lobulo-alveolar development [66]. These results indicate that MG development or at least the maintenance of a mammary functional structure depends on the presence of specific nutrients in the diet.

Stair-step energy restriction treatment is a repeatable two-steps diet, consisting of an alternative schedule energy restriction for few days, followed by realimentation to feed offered *ad libitum*. This feeding strategy is commonly used in animal breeding especially in bovine, during the life of animal, to program the onset of puberty [67]. Studies using stair-step diets have been realized to assess the effects of this nutritional strategy on further MG development and lactation. For this purpose, rodents and pigs have been mainly used as experimental models.

In rats, stair-step restriction did not affect mammary cellular composition, but it reduced fat deposition in the MG at mid-pregnancy [68]. During lactation, stair-step diet increased β-casein mRNA accumulation and milk protein secretion and stimulated mammary cell proliferation, thus improving lactation performance [69,70]. A strategy of compensatory restriction, including a period of caloric restriction of 40% in early pregnancy followed by free feeding, showed an enhanced milk production characterized by an elevated mammary cell proliferation rate in late pregnancy and early lactation. These effects were persistent, since a decrease in the regression of mammary cells displayed by reduced caspase-3 enzyme activity was observed over two consecutive lactations [71]. Furthermore, an extreme experimental design was performed on lactating rats, starved for 48 h and refed on a high-carbohydrate diet for an additional 48 h. Starvation stopped milk secretion, which resumed shortly after refeeding. Three lipogenic enzymes (FA synthase, glucose 6-phosphate dehydrogenase, and malate dehydrogenase) decrease in the MG during starvation, but their levels were restored 48 h after refeeding [72], indicating some flexibility in the diet-induced processes in the MG.

In pigs, few data are available. Before puberty, diet deprivation followed by an *ad libitum* allowance feeding was reported to lead to impaired MG development in gilts, characterized by a lower amount of parenchymal tissue, fat, and protein contents [20]. However, when feed-deprivation was carried out from puberty and was followed by over allowance, an increase in milk yield and ß-casein content was observed in pregnant mammary tissue, thus confirming that puberty is a critical window for nutritional regulation of MG development [73]. When a similar dietary stair-step treatment was applied in sows after puberty during the only growing-finishing period, mammary parenchymal tissue mRNA composition analysis at late pregnancy showed a reduced protein/DNA ratio accompanied by a high STAT5B mRNA level [20,74]. The reduction in parenchymal tissue induced by the stair-step diet was higher after puberty than in late pregnancy, when a reduction in protein synthesis was observed, suggesting that during pregnancy, detrimental dietary effects may be reversed.

## 5. Effects of Isoenergetic Modifications

Maintaining a controlled energy intake for global health and here, for optimal breast development, throughout life is of utmost importance. However, the contribution of specific components in varied diets should not be underestimated. Indeed, an excessive intake or a deficiency of certain nutrients may not affect global energetic intake but can have important effects on the health of the individual and, by extension, on the functionality of certain organs including MG [75].

Dietary FAs can modulate mammary development and subsequent milk quality. The role of specific FAs, outside the context of energetic changes, has been extensively studied in ruminants because of their impact on milk composition. However, rodents are also model of choice for studying their influence on mammary tissue development including underlying mechanisms [76]. In early life, N-3 polyunsaturated FA (PUFA) consumption plays a major role in the structural development of mammary tissue during puberty [77]. Polyunsaturated FAs are also involved in mechanisms responsible for rapid ductal elongation and branching as well as in increased terminal end buds number [78]. During pregnancy, MGs of rats fed tuna oil (n-3 enriched oil), as a source of dietary polyunsaturated FAs compared to those of rats fed an isocaloric diet containing corn oil (n-6 enriched oil) showed an increase in the size of cytoplasmic lipid droplets and an unusual formation of a multilayer epithelial sheet with a high cell proliferation rate, thus highlighting the influence of n-3 polyunsaturated FAs on mammary epithelial differentiation during pregnancy and lactation [79,80].

Changes in consumption of specific FAs have been used to decipher the molecular pathways involved. Oleic acid a monounsaturated FA, is mainly found in olive oil [81]. Mice fed a diet containing 2% oleic acid during the peripubertal period showed enhanced growth of the MG characterized by an increased number of terminal duct ends and ductal branching, together with enhanced expression of CD36, which is involved in the PI3K/Akt pathway, and cyclin D1, which is involved in cell proliferation [82]. Similar stimulating effects on mammary duct development were observed in mice fed 1% lauric acid, a saturated FA found mainly in coconut oil [83], including activation of the PI3K/Akt pathway and enhanced expression of cyclin D1 and G protein-coupled receptor 120 [83]. Beneficial effects were also observed in lactation including increase in cellular proliferation and casein synthesis, related to an activation of the PI3K/Akt signaling pathway [84]. However, when mice were fed a 2% lauric acid isocaloric diet, a reduction in terminal end buds’ number and ductal branching was observed. Moreover, inhibitory effects of certain FAs such as stearic acid have been reported, such as suppression of mammary development in pubertal mice by inhibiting the PI3K/Akt signaling pathway through activation of G protein-coupled receptor 120 [83]. A decreased mammary lobulo-alveolar development in late pregnancy impairing subsequent lactation was observed when mammary synthesis of arachidonic and docosahexaenoic (DHA) acids was modified following an isocaloric hypoproteic diet [85], thus highlighting the importance of the quantitative balance of each dietary component as well as that of the physiological stage [83].

Amino acids, present in both free and peptide-bound forms, are the most abundant organic nutrients in the milk. A modification in the protein content of the diet may thus strongly impact mammary development and milk composition. Numerous studies have been led using rodents and pig animal models.

A protein restriction without modifying the total energy intake, has shown effects on the structural modification during mammary development in rodents, by decreasing the development of mammary epithelial tissue associated with a lower number of alveoli and a higher adipose tissue representation in early and mid-lactation. In addition, epithelial cells exhibited reduced nuclei and cytoplasm [85]. These effects are accompanied by changes in milk nutrient content (lower protein and higher FA) and a lower milk production [86]. Similar features have been reported with a specific arginine depleted diets, which had a deleterious impact on MG development and milk protein synthesis in lactation, demonstrating that poor protein intake induces deleterious mammary development in late pregnancy and may even have a negative impact on subsequent lactation [87]. Isocaloric diets containing whey proteins as protein sources led to increased mammary phosphatase and TENsin homolog (PTEN) expression in rats and therefore to enhanced mammary cell differentiation, since PTEN plays a critical role in controlling cell survival through antagonistic effects on the PI3K/Akt pathway [88,89].

Contrary to data observed in rodents, modifying the total protein intake without modifying the energy intake did not show any effect on mammary development during late pregnancy in gilts [52,90]. In contrast, the qualitative decrease in the dietary protein fraction during pregnancy leads to functional modifications of mammary epithelial cells. Dietary valine supplementation during late pregnancy had strong effects on porcine MG structure. Valine is transported at the highest dose in the MG, suggesting that this limiting amino acid may play a crucial role in physiological metabolism in the MG [91]. Dietary valine supplementation promotes increased lumen area of alveoli, as well as DNA, RNA and total protein content in mammary tissue as well as protein, fat, and free amino acid content in the colostrum of gilts in early lactation [91]. Moreover, in the same species, increasing dietary valine allowance transitorily increased mammary expression of Cyclin D1 and activated phosphorylated mTOR, P-S6, and p-4EBP, at day 1 of lactation [3,91]. Taken together, these data suggest that valine supplementation may promote mammary epithelial cell proliferation. Studies have also reported that lysine and arginine supplementation during late pregnancy can improve lactation performance in sows [92,93,94]. Tryptophan supplementation induced the biosynthesis of FA synthase, lactose synthase, and β-casein in porcine mammary epithelial cells and increased milk yield and calcium concentration, which is correlated to increasing expression and activities of binding proteins and kinases involved in calcium metabolism [95]. α-ketoglutarate is a key molecule in the Krebs cycle and maintaining an optimal α-ketoglutarate level for cells is a prerequisite to many physiological processes such as proliferation and differentiation. Jiang et al. [96] showed that α-ketoglutarate contributes to improvement in lactation performance by stimulating protein synthesis via mTOR activation. Dietary supplementation with α-ketoglutarate in isocaloric diets in sows induced an increase in lactose and calcium concentrations in milk [97].

Carbohydrates are known for their roles in providing substrate and energy to developing and further lactating MG. The sources and amounts of dietary carbohydrates during pregnancy have been studied extensively in animal models with regard to the risks of gestational diabetes and its consequences on the human health [75]. The impact of dietary carbohydrate restriction, mimicking isoenergetic diets containing different levels of glucose, was described in rats fed a diet without glucose. Absolute or severe colostrum-restricted milk production leading to pup death has been observed [98,99]. A reduced dietary glucose level, but higher than 5% in the food ration over a period covering gestation and lactation, reduced mammary size but did not strongly modify MG content regarding protein, fat, or glycogen [99], suggesting that altered lactation performance was not due to impaired MG content but partly to reduced MG size. However, rats fed a restricted glucose diet produced milk with a lower fat concentration but unchanged protein and lactose concentrations [99]. Taken together, these data show that glucose intake is crucial for mammary function during pregnancy and lactation and that at least 25% glucose intake is needed to produce milk with an optimal fat concentration.

Isoenergetic diets have also been used to assess effects of single nutritional components, which are not related to a calorie intake or deprivation, such as vitamins, minerals, and natural steroids. When a whole-food-fruit-rich diet was used as a source of vitamin A in rats, alveolar development was reduced and characterized by a low number of acini per lobule [100]. Furthermore, acidic protein expression decreases and tends to be undetectable in the MG of rats fed β-carotene-rich fruits and vegetables, mimicking vitamin A supplementation, at adult onset [100]. During lactation, increased food intake provides a high vitamin A level, which is associated with increased redistribution in the mammary tissue and thus in the secreted milk [101]. Concerning minerals, a few effects have been reported on mammary development after diet-induced iron deficits, except an increased density of terminal end buds together with decreased differentiation and impaired epithelial cell growth in rats [102,103]. Mice fed a zinc-deficient isoenergetic diet showed alterations such as aberrant collagen deposition and ductal and stromal hypercellularity in the mammary microenvironment. These modifications impaired ductal expansion by collapsing the lumen and disorganizing the mammary epithelium [104]. Dietary zinc deficiency also severely compromised the MG structure by impairing mammary architecture, reducing the number of alveoli in lactation and increasing epithelial cell apoptosis [105]. Moreover, milk production was drastically decreased as well as the zinc concentration in the milk. Fat and lactose contents in milk were not affected by zinc deficiency, although milk protein concentration and distribution were modified [105]

Studies conducted on different species have begun to decipher the role of phytoestrogen treatments on mammary development, but poor documentation exists concerning the impact of dietary consumption of phytoestrogens [106,107]. Flaxseed is high in α-linolenic acid, and its consumption can produce mammary structure modifications in rats: female rats fed a 5% flaxseed diet showed an antiestrogenic-induced reduction in terminal end buds due to atrophy, while a 10% flaxseed diet produced estrogenic effects that also resulted in terminal end buds’ reduction but due to lack of differentiation [108], demonstrating strong dose-dependent effects. Feeding rats with 10% flaxseed during pregnancy and lactation led to a decrease in terminal end buds and an increase in alveolar buds [108,109]. Lactation therefore appears to be a critical period for enhancing the differentiation of terminal end buds to alveoli buds by dietary phytoestrogens [110]. Genistein is a major component of soy and one of the most consumed phytoestrogens worldwide. In gilts, dietary supplementation with genistein during the growing phase led to hyperplasia of the mammary parenchymal tissue after puberty, without changes in the expression of genes involved in the estrogenic signaling pathway in the MG [111]. In contrast, prepubertal dietary supplementation with flax from different sources such as seeds, meal, or oil in gilts had a very few consequences on mammary development, except for an increase in dry matter mammary content [112]. Alkaloids, nitrogenous organic compounds produced by a large variety of organisms, have strong physiological and toxic effects on the host [113]. In sows, MG development is impacted following a sorghum-ergot-rich diet, since the alkaloid present in sorghum ergot interferes with the release of prolactin to induce milk production. High rates of sorghum ergot impaired lactation, even causing agalactia, when used at 1.5% [114]. Taken together these results highlight the importance of components, non-essential regarding their energy value. Moreover, the dose and source of the components as well as the timing and the physiological stages of their effects are of utmost importance for mammary development and subsequent lactation.

It is now well established that diet-induced modifications in morphology and function of the MG are correlated with the nutritional value of milk. Given the importance of milk consumption in human and animal nutrition and health, ruminants are the most relevant model to assess the dietary influence of nutrients on mammary development in lactation and in resulting milk composition.

## 6. Nutritional Regulation of Gene Expression Involved in Milk Component Synthesis in Ruminants

After the period of MG development, the parturition is the signal of the commissioning of the mammary epithelial cells. During lactation, these cells synthesize and secrete large quantities of specific milk components, including proteins, lactose, and lipids, which have a considerable effect on the nutritional, technological, and sensorial properties of milk products. Milk production and composition are affected by genetic, nutritional, physiological, and environmental factors. Nutrition in particular has a considerable effect on the composition of milk lipids [115], whereas the composition of the protein fraction is generally only marginally affected by this factor [116]. Due to human consumption of ruminant milk and the influence of milk composition on its nutritional quality, the nutritional regulation of milk components in the MG is mainly studied in ruminants (bovine, caprine, and ovine species). Putting aside the role of the rumen in nutritional responses in ruminants, we will focus this review on the gene expression in the MG.

## 7. Effects of Lipid-Supplemented Diets on Ruminant Mammary Gene Expression

**Figure 2:**
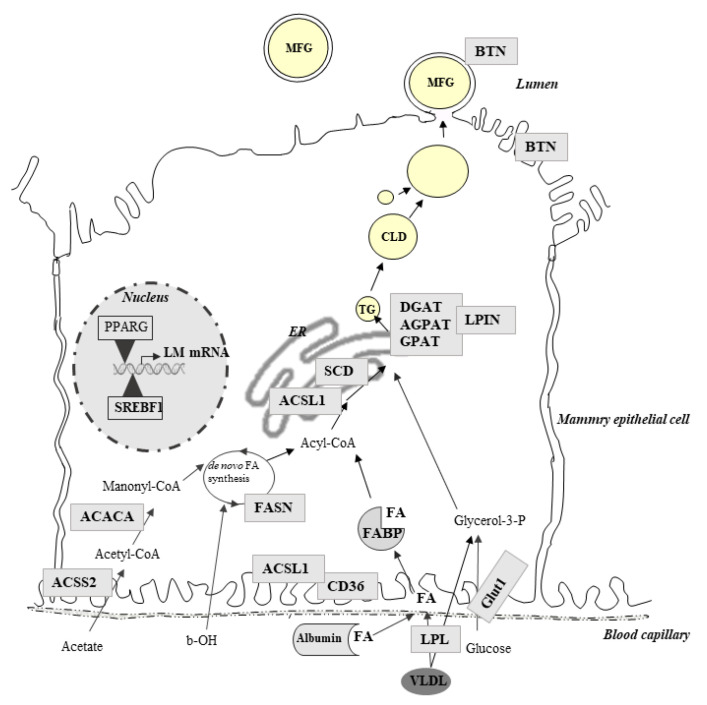
Milk fat synthesis in ruminant mammary epithelial cells. Key proteins are in gray boxes. ACACA: acetyl-CoA carboxylase; AGPAT: acyl glycerol phosphate acyl transferase; ACSL: acyl-CoA synthetase long chain; ACSS2: acyl-CoA synthetase short-chain family member 2; BTN: butyrophilin; CD36: cluster of differentiation 36; CLD: cytoplasmic lipid droplet; CoA: coenzyme A; DGAT: diacyl glycerol acyl transferase; ER: endoplasmic reticulum; FA: fatty acid; FABP: fatty acid binding protein; FASN: fatty acid synthase; Glut 1: glucose transporter-1; GPAT: glycerol-3 phosphate acyl transferase; LM mRNA: lipogenic gene mRNA; LPIN: phosphatidate phosphatase LPIN1; LPL: lipoprotein lipase; MFG: milk fat globule; PPARG: peroxisome proliferator activated receptor γ; SCD: stearoyl-CoA desaturase; SREBF1: sterol regulatory element binding transcription factor 1; TG: triglyceride; VLDL: very low-density lipoprotein.

Milk FAs are largely responsible for the nutritional quality of milk. Indeed, some FAs are suspected to have negative effects (e.g., saturated FAs or C18:2n-6) if they are consumed in excess, whereas others may have positive effects (e.g., cis-9 C18:1, C18:3n-3, and n-3 polyunsaturated FAs). In ruminants, nutrition is one of the major factors that can improve milk FA composition. Thus, researchers have reported many studies aiming to improve milk FA composition via ruminant nutrition. One of the strategies to improve the milk FA profile is lipid supplementation. However, the diet has different effects depending on the nature of the forage, the forage-concentrate ratio and the lipid supplements used [117]. To precisely regulate the composition of milk, studies to better understand the mechanism underlying these effects have been carried out. Lipid supplements affect mammary lipid metabolism partly through modulation of the expression of genes involved in lipid metabolism. Indeed, FAs in milk come almost equally from nutrition (long FAs) and de novo synthesis (short FAs) in the MG. Thus, molecular studies by analyses of the expression of lipogenic candidate genes in ruminant MG have been carried out (Figure 2).

In lactating cows, the effects of many sources of lipids on MG gene expression were studied using a candidate gene approach. In cows, supplementation of a starch rich with vegetable oils or with marine lipids leads to milk fat depression [118]. Milk fat depression diets reduce the fat content by up to 50% and change the FA composition of the milk with a decrease in the synthesis of short-chain FAs [118,119]. Ruminal biohydrogenation of unsaturated FAs occurring with a milk fat depression diet leads to an increase in the production of trans-10 C18:1 and trans-10, and cis-12 conjugated linoleic acid (t10, c12-CLA), which is known to inhibit de novo mammary FA synthesis [120]. Studies of MG gene expression associate the consumption of fish oil with a reduction in lipogenic mRNA abundance. In particular, acetyl-CoA carboxylase (*ACACA*) and FA synthase (*FASN*), both of which are involved in de novo synthesis, were downregulated by the consumption of 1.5% to 3% protected fish oil [119]. Similar results were obtained using supplementation with plant oils (linseed or sunflower oils, each at 2.7% of the basal diet) and DHA-rich algae (0.4% of the basal diet), resulting in milk fat depression. Consistent with milk fat depression, the analyses of lipogenic gene expression showed a downregulation of *FASN* in MG [121]. Vegetable oils without marine oil also affect the expression of genes involved in de novo FA synthesis. Thus, the expression of *ACACA* and *FASN* was decreased by supplementation of a basal diet (25:70 forage:concentrate ratio) with soybean oil (5% basal diet), a rich source of C18:2 cis n-6 [122]. A reduction in the expression of the *FASN* gene was also detected by comparing a control and a low forage with a high oil diet including 3% soybean and 1.5% fish oil, associated with a decrease in milk fat yield and content [123]. Reductions in the expression of *ACACA*, *FASN*, glycerol phosphate acyltransferase (*GPAT*), and acylglycerol phosphate acyltransferase (*AGPAT*), which are involved in triglyceride synthesis, were observed to be associated with milk fat depression obtained with a high concentrate/low forage diet without marine oil supplementation [124] (Figure 2). In addition, a positive correlation was observed between milk fat content and the gene expression of *ACACA* and *FASN*, suggesting that fish oil reduces the milk fat percentage by inhibiting lipogenic enzymes [119].

Dairy ewes are less inclined to milk fat depression [125] except with diets supplemented with marine lipids [126]. As in cows, the inclusion of fish oil (2.4%) reduced the milk fat concentration, which is associated with a decrease in the mRNA abundance of *FASN*, acyl-CoA synthetase short-chain family member 2 (*ACSS2*), and lipin 1 (*LPIN1*) [127,128].

Conversely, milk fat depression is not usually observed in goats under dietary conditions inducing milk fat depression in cows [129], suggesting that the effects of diets with fish oil or rich in starch were different according to the ruminant species. Thus, supplementation with sunflower seed oil (60 g/day) and fish oil (30 g/day) plus additional starch (102 g/day) induced limited changes in the mRNA abundance of genes involved in lipid metabolism [130]. To decipher the different responses to marine lipids between cows and goats, a direct comparison was performed using marine algae powder (1.5%) [131]. However, milk fat content and FA secretion were not accompanied by a modification of the expression of key lipogenic genes in MG, except for 3 transcription factors (*PPARA*, *INSIG1*, and *SP1*). Due to the role of t10, c12-CLA in the induction of milk fat depression, a CLA-induced milk fat depression supplement was studied in goats, showing a decrease in the expression of *FASN*, *ACACA*, *LPL*, *ACSL1*, *DGAT2*, *BTN1A1,* and *CD36* in MG [132]. In addition to milk fat depression diets, the effects of vegetable-supplemented diets on caprine lipogenic gene expression have different effects on milk fat content than the effects that are observed in cows. Indeed, milk fat yield and content were increased with diet supplementation with soybeans (3.8%) [133] and linseed (11.2%) [134] oils. However, these increases were not accompanied by a modification of key lipogenic gene (*ACACA*, *FASN,* and *LPL*) expression in MG.

Due to the nutritional controversial effects of saturated FA, several studies have focused attention on the regulation of *SCD* gene expression. In ruminants, as observed in mice fed a high-fat diet, *SCD1* is one of the most highly expressed genes and appears to be key during milk fat synthesis [135], whereas *SCD5* is less expressed in MG. The regulation of *SCD* gene expression at the mRNA level was governed by the forage: concentrate ratio and the nature and level of polyunsaturated FAs in the diet. We can also suspect that, as described above in mice, *SCD1* gene expression is regulated by epigenetic mechanisms [41]. The expression was downregulated by diets inducing a milk fat depression [124] and with protected fish oil [119] or DHA-rich algae [121]. A reduction in *SCD* gene expression was also detected with soybean oil supplementation, which was not accompanied by milk fat depression [136]. Conversely, an increase in *SCD* gene expression was observed with diets supplemented with fish and soybean oils [137]. This increase is in line with the increase observed in mice fed a high-fat diet as described above [40]. In goats and ewes, the expression of *SCD1* is not affected by the addition of fish oil (30 g/day) and starch to a diet containing sunflower seed oil (60 g/day) [138]. The absence of modification of *SCD1* gene expression was also observed in mammary ewes after the addition of fish oil or t10, c12-CLA to a forage: concentrate ratio 40:60 basal diet [127].

The differences in responses to lipid supplements emphasize the complexity of the regulation of the expression of genes involved in lipid metabolism. To decipher the molecular mechanism underlining this regulation in the MG, several studies have examined the expression of transcription factor genes. A central role for the transcription regulator sterol regulatory element binding factor 1 (*SREBF1*) has been outlined as a mediator of FA effects [134]. A combination of DHA-rich algae and sunflower or linseed oils led to a decrease in milk fat yield with a decrease in *SREBF1* and *FASN* gene expression [121]. In addition, fish oil or rumen-protected microalgae supplementation also led to a decrease in the expression of *SREBF1* and sterol regulatory element-binding protein cleavage-activating protein (SCAP) but without modification of the expression of the *FASN* gene [139]. The SREBF1 signaling pathway is not the only pathway to play a crucial role in the regulation of the expression of lipid metabolism genes. Gene network analyses highlighted a pivotal role for the concerted action of two peroxisome proliferator activated receptor γ (*PPARG* and *PPARGC1A*) and insulin-induced gene 1 (*INSIG1*) [135]. The increase in secretion of de novo synthesized FAs by indoor marine (fish or microalgae) supplementation compared with grazing conditions was accompanied by an increase in mammary *PPARG* mRNA abundance [139]. The expression of *PPARG* was also increased by a t10, c12-CLA-rich diet with a milk fat depression in ewes [127]. The PPAR pathway was also modulated by a milk fat depression diet containing marine algae powder in the goat MG [131]. However, in this study, the expression of *PPARG* was not affected, but *PPARA* was decreased, as well as *INSIG1*, without modifications of the expression of lipogenic genes such as *ACACA* or *FASN*, possibly due to the large network of regulators. Indeed, e.g., *INSIG1*, an endoplasmic reticulum membrane protein, regulates *SREBP* via its interaction with *SCAP*, which regulates the activation of *SREBP1*. This link between the two regulatory pathways (*SREBF* and *PPAR* pathways) highlights the complexity of the nutritional regulation mechanisms of lipid metabolism in the MG of ruminants. In addition to these key regulators, others such as *LPIN1*, which acts as a nuclear transcriptional coactivator for *PPARGC1A/PPARA* to modulate lipid metabolism gene expression, have also been reported. In addition, thyroid hormone-responsive SPOT14 (THRSP) was identified as a key component of the t10-, c12-CLA regulation of bovine milk fat synthesis [123,129]. Invernizzi et al. [137] showed that fish and soybean oils increased *SCAP* expression, whereas the *SPOT14* mRNA levels dropped but returned rapidly to basal levels in Holstein cows, consistent with the lack of change in *SPOT14* expression observed in ewes after 54 d of feeding a diet containing marine algae [140].

More recently, to obtain an overview of MG function and responses to diets, the available transcriptomic tools for ruminants have been used, allowing a more global understanding. The first transcriptomic studies used microarray analyses. The recently introduced high-throughput RNA sequencing (RNA-Seq) approach provides a more sensitive method that does not require *a priori* information. However, the specificity of MG, which contains up to 80% casein mRNA during lactation, must be considered to define the sequencing criteria. In cows, transcriptomic studies using microarrays showed that vegetable oil supplementation often affected the expression of genes involved in the remodeling of the MG, which might be accompanied by modifications of milk FA profiles. Thus, supplementation with unsaturated FAs (using either unprotected rapeseed, soybean, linseed oils, or a proportional mix of these) induced a reduction in short chain FAs, reflecting a decrease in de novo FA synthesis [141]. This study also showed a modification of the expression of genes involved in molecular transport, lipid and protein metabolism, and nutrient metabolism, as well as of sets of genes involved in cell development and remodeling, apoptosis, and the immune response. These last pathways were predominantly downregulated and negatively correlated with milk trans-FA concentrations [141]. Studies on different plant oil supplementation methods showed different effects between linseed oil (rich in α-linolenic acid, 5%) and safflower oil (rich in linoleic acid, 5%) supplementation on bovine MG gene expression [142]. Indeed, linseed oil supplementation affected the expression of more than 1000, whereas safflower oil modified the expression of less than 200. However, both diets led to a decrease in the milk fat percentage, with a decrease in saturated FAs, including short FAs, which was due to the decrease in de novo synthesis in MG.

**Figure 3:**
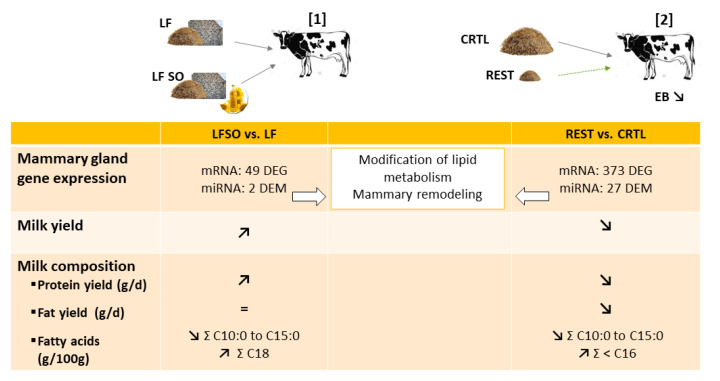
Effects of sunflower supplementation and restricted diet on milk and mammary gene expression. Reprinted with permission from ref. [1] Copyright 2019 Cambridge Core; Reprinted with permission from ref. [2] Copyright 2021 MDPI. CTRL: control diet; DEG: differentially expressed mRNA; DEM: differentially expressed microRNA; EB: energy balance; LF: low forage; LFSO: low forage with 4% of sunflower oil; REST: restricted diet to covered 50% net energy for lactation.

The interaction between the nature of the lipid and the basal diet also had different effects on gene expression. Thus, supplementation with sunflower oil (4%) in a low forage-based diet showed a higher amplitude of milk composition and mammary transcriptome responses than supplementation with whole intact rapeseed (14%) in a high forage diet [143]. Rapeseed supplementation did not change the transcriptome of MG, whereas the expression of 49 genes was altered by sunflower oil compared to the low forage diet, in agreement with the observed decrease in the milk fat content with sunflower oil supplementation (Figure 3). However, the expression of the key lipogenic genes (e.g., *ACACA*, *FASN*, and *SCD1*) did not significantly change, although a decreasing trend was observed for *FASN*. Similar supplementation diets were studied in caprine mammary transcriptomes and showed lower responses in goats than the responses observed in cows [144]. In goats, milk fat content was higher after intact rapeseed or sunflower oil supplementation, whereas no change was detected in the transcriptomes in the MG. As previously reported, the effects of the same supplementations on mammary transcriptomes were lower than the effects observed in cows. However, the global expression profile allowed the samples to be classified according to diet, suggesting too weak differences in the expression of each gene but modifications detected by overall analysis.

**Figure 4:**
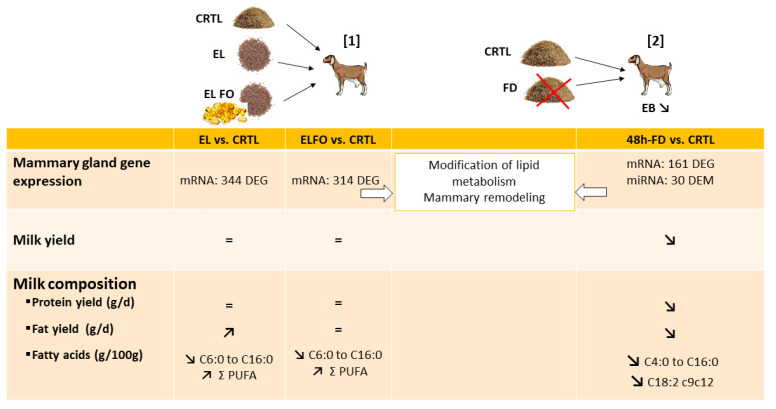
Effects of extruded linseeds (EL) alone or in combination with fish oil (ELFO) supplementation and of 48-h feed restriction (REST) in lactating goats on milk and mammary gene expression. Reprinted with permission from ref. [1] Copyright 2019 Cambridge Core; Reprinted with permission from ref. [2] Copyright 2021 MDPI. CTRL: control diet; DEG: differentially expressed mRNA; DEM: differentially expressed microRNA; EB: energy balance.

To enlarge the analyses of the different responses to supplementation between cows and goats, fish oil supplementation was also studied in goats (Figure 4). Thus, the effects of extruded linseeds (530 g/d) alone or in combination with fish oil (extruded linseed: 340 g/day and fish oil: 39 g/d) supplementation on caprine mammary gene expression were studied [145]. The extruded linseed alone or in combination with fish oil diets influenced the expression of 344 and 314 genes (with 76 in common), respectively, in comparison with the control diet. Among them, more than 20 genes were involved in lipid metabolism and transport class. Eight common genes (*ALDH3B1*, *ALDH18A1*, *DGKD1*, *ENPP1*, *IL7*, *NSMAF*, *PI4KA,* and *SERINC5*) were downregulated by these two treatments. The extruded linseeds with fish oil supplementation diet modulated the expression of *PPARG* and *SREBF1*, known to regulate lipogenic gene expression, as discussed above. In addition, two regulatory networks centered on the estrogen receptor (*ESR1*) and a transcription factor (*SP1*) were identified in the diets supplemented with extruded linseeds alone or in combination with fish oil, respectively, compared to the control diet. In addition, these supplementations altered protein and amino acid metabolism without changes in major milk protein secretion. Furthermore, the first and second pathways altered by the extruded linseed alone or in combination with fish oil diets, respectively, compared to the control diet are the PI3K/Akt signaling pathway, which is in line with results obtained in mice fed a diet containing oleic acid as described above [82]. In addition, the two supplementations induced large changes in milk FA without effects on mRNA linked to lipid metabolism [145]. In addition, lipid supplementation in goats might affect mammary remodeling [145], as suggested in bovine MG [141].

In ewes, the effects of inducing milk fat depression diets were also studied using milk somatic cells. Indeed, mammary epithelial cells are exfoliated from the mammary epithelium and are an alternative sample to study MG expression [146]. Using RNA-seq, a comparison between two diets inducing milk fat depression (CLA vs. fish oil diets) showed 653 differentially expressed genes (DEGs) in milk somatic cells according to the supplementation, whereas fish oil supplementation, compared to the control diet, altered the expression of 237 genes [147]. Among the 55 genes communally modulated by both milk fat depression diets compared to the control diets, *ACACA*, *AACS*, *ACSS2*, *ACSS3,* and *FADS2*, which are involved in FA synthesis, were downregulated, which is in line with the milk fat depression.

All these studies pointed out the complexity of the lipid supplementation effects and identified mainly regulated genes that were not considered in the past as candidate genes due to their function. Several studies also highlighted the effects on mammary remodeling or structure, raising the question of the effects of such supplementation on lactation throughout the life of livestock animals. To elucidate the complexity of the regulation of the coding gene expression profile, miRNome analyses were undertaken. Indeed, microRNAs (miRNAs) are small noncoding RNAs considered regulators of at least 60% of coding genes by base pairing mRNAs, inducing their degradation or inhibiting their translation [2,148]. Many studies have considered the effects of one miRNA using in vitro analysis (such as bovine or goat mammary epithelial cells). In this review, we will focus our attention on in vivo studies. First, in livestock, due to their economic importance, lactation function has been investigated mostly in ruminant species, and consequently, ruminant mammary miRNomes have been established [149,150,151,152]. To better understand the coding gene regulation in the MG in response to lipid supplementation, miRNAs analyses using RNA-Seq were performed in lactating cows supplemented with sunflower oil (4%) supplementation [153] (Figure 3) and linked with mRNA transcriptomic data from the same trial [143]. Although this study was performed using a limited number of cows, the expression of two miRNAs (*miR-20a-5p* and *miR-142-5p*) was modulated by sunflower oil supplementation. Bioinformatics analyses suggested that they could target genes identified to be differentially expressed [143]. In particular, they are both predicted to target the *ELOVL6* gene involved in lipid metabolism [153]. Similarly, the decrease in milk fat percentage and saturated FA induced by diets supplemented with linseed oil (5%) and safflower oil (5%) was simultaneously observed with an alteration of the expression of 14 and 22 miRNAs, respectively [154]. Seven miRNAs were common between these two analyses. Their predicted targets are involved in lipid metabolism, suggesting their potentially important role in the synthesis and secretion of milk components.

## 8. Effects of Feed Restriction and Deprivation on Ruminant Mammary Gene Expression

Early lactation is a classical situation of physiological undernutrition and negative energy balance because feed intake increases at a slower pace than the requirements for milk production. The prioritization of nutrient partitioning to MG and milk synthesis leads to mobilization of body protein and fat reserves and modifications in milk protein, fat and FA composition [155,156]. Complex homeorhetic and homeostatic adaptations are required to directly limit nutrients toward the MG and support milk synthesis during early lactation [156]. In addition, natural negative energy balance also exists in tropical and Mediterranean regions, where seasonal weight loss was observed in ruminants, limiting production, especially milk production and yield [157]. Indeed, in these areas, long dry seasons lead to poor pastures with low nutritional value. During the dry season, ruminants, particularly those raised in extensive production systems, lose approximately 30% of their body weight [158,159]. In addition, it is now well accepted that one of the main effects of climate change will lead to food availability. To mimic the state of negative energy balance, experimental feed restriction or deprivation models are used to induce rapid metabolic changes and alterations in milk yield and composition as well as in the FA profile, with the hypothesis that restriction would induce corresponding changes in the function of the MG [160,161,162,163,164]. Thus, the use of restriction models inducing a controlled negative energy balance in ruminants is very important to better understand the mechanisms underlying changes in milk production and composition. Better knowledge of these mechanisms could improve the detection of nutritional deficiencies in ruminants and minimize their effects. Complementary approaches were used to decipher the molecular mechanisms associated with natural or induced negative energy balance in MG. The effects on the expression of genes were investigated through targeted studies of genes involved in mammary lipogenesis using RT-qPCR and global analyses using transcriptomic and proteomic analyses.

Studies were first focused on the effect of negative energy balance on the expression of a few genes encoding the key lipogenic enzymes in MG. Bionaz and Loor [165] found that in bovine MG, lipogenic gene (*ACACA*, *FASN*, *LPL*, *SCD*, *PPARG,* and *SREBPF1*) expression decreased during the natural negative energy balance period of early lactation (day 15 of lactation) compared to mid-lactation (day 60 of lactation). These gene expression decreases are associated with a lower fat yield, suggesting an important role for these genes in the maintenance of milk synthesis. A significant interaction between stage of lactation and parity was observed due to a lower expression of *FASN* and *ACACA* in MG of Holstein primiparous cows in early lactation [166]. This study also highlighted lower expression of the *SCD* gene at early lactation compared to peak and late stages of lactation. Regarding studies of negative energy balance induced by feed restriction, in mid-lactating Holstein dairy cows, a restriction for 4 days at 60% ingestion (compared to control diet) induces a downregulation of the expression of mammary lipogenic genes [163]. Indeed, feed restriction decreased the expression of *LPL* and *FABP3*, which are involved in FA transport; *ACACA*, *FASN*, and *SCD1*, which are involved in de novo FA synthesis and desaturation; *GPAT*, which is involved in triglyceride synthesis; *FABP3*, which is involved in intracellular transport; and *PPARG*, which is involved in the regulation of transcription. Similar results were observed in Friesian cross-bred dairy sheep. The *ACACA*, *FASN*, *LPL,* and *SCD* mRNA levels in MG were reduced in underfed sheep (at 70% of the animal’s requirements for 60 days) compared with control sheep [167]. The mRNA of *ACACA* and *FASN* decreases by restriction are in accordance with the sharp decrease in the synthesized de novo (short- and medium-chain FAs) FAs in milk and the decrease in milk fat yield [163,167,168]. The same relationship between the reduction in *FASN* mRNA in the MG of lactating goats and the decrease in medium-chain FA in their milk has been found by Ollier et al. [144] when the goats were subjected to 48 h of food deprivation as in rats [72]. This treatment caused a drop in milk production and component secretion in goats [157,169,170]. A significant reduction in the mRNA levels of *ACACA*, *FASN*, *LPL*, and *SCD1* was also observed in the MG of underfed dairy goats compared with the respective overfed goats [171]. These data, in the bovine, the ovine, and the caprine MG, suggest a regulation of these genes at a transcriptional level by nutrition and indicate that the decrease in nutrient availability may lead to a lower rate of lipid synthesis.

The development of high-throughput tools such as microarrays and, more recently, high-throughput sequencing and proteomics analyses allows the study of several thousands of genes and provides the possibility to better decipher the molecular mechanisms of MG response to negative energy balance.

The transcriptome was altered in bovine MG during the negative energy balance period [172,173,174]. Thus, feed restriction (at 60% of calculated net energy for lactation requirements) for 5 days resulted in 278 [172] and during 6 days in 374 [174] (Figure 3) differentially expressed genes in the MG of mid-lactation Holstein cows. Among 180 upregulated differentially expressed genes observed, 8 genes (*APP*, *AKT1a*, *BTN1A1*, *GPAT*, *LPIN1a*, *PPAP2C*, *PRKAA2*, *and VLDLR*) were involved in lipid metabolism, and 14 were involved in molecular transport and corresponded to the most enriched molecular functions [172]. The 98 downregulated genes were associated not only with cell growth and proliferation but also with cell death [172]. After 6 days of feed restriction, among the 374 mammary differentially expressed genes between restricted and control diets, 120 were upregulated and 254 were downregulated, including 28 genes involved in lipid metabolism and 33 in endothelial cell proliferation [174]. The downregulation of genes involved in cell proliferation in both studies suggests a modification in the mammary structure. In addition, the downregulation of *ACACA*, *FABP3,* and *LPL* reported by Billa et al. [174] was in accordance with the decrease in milk fat yield as with the reduction in their expression after 4 days of restriction in MG from cows fed at 60% of their *ad libitum* dry matter intake as described above [163]. The downregulation of *ACSL1*, *FABP3*, and *LPIN1* in mammary tissue of dairy cows [174] is in line with the decrease in their expression during the natural negative energy balance period of early lactation (day 15 of lactation) compared to mid-lactation (day 60 of lactation), which is associated with a lower fat yield [165]. Similar results were shown in the MG of early lactation Holstein cows in response to undernutrition after an intramammary 24-h lipopolysaccharide (LPS) challenge [173]. Indeed, microarray analyses identified 33 differentially expressed genes in response to diet dilution (at 48% barley straw and dry matter basis) for 4 days compared to control cows: 19 genes were upregulated and 6 were downregulated. Most of the differentially expressed genes in MG are involved in metabolism, including the regulation of FA and glucose metabolism (*CPT1A*, *PDK4*, and *PFKFB4*), carnitine shuttle (*SLC25A20*, *CPT1A*, and *SLC25A34*), regulation of cellular ketone metabolic process (*PDK4*), and the key genes in those processes. These results suggested that restriction modified mammary metabolism, specifically the β-oxidation process.

In goats, 48-h food deprivation also alters the mammary transcriptome [170]. Indeed, 48-h food deprivation, compared to the control diet, affected the expression of 161 genes in the MG (Figure 4), including those coding for lipogenic enzymes and major milk proteins [170]. Most of these genes (88%) were downregulated, particularly those involved in lipid, protein, and lactose metabolism, as a stress response of MG to the lack of nutrient supply, suggesting an early involution. The decrease due to food deprivation in the expression of *LALBA*, *CSN1S1*, *CSN1S2*, *CSN2*, and *LGB* genes encoding 5 of the 6 main ruminant milk proteins [175] is associated with a sharp fall in milk protein yield [170]. The downregulation of *LALBA* expression, which is involved in lactose synthesis, is also in agreement with the drop of milk lactose yield. Furthermore, food deprivation led to an abrupt downregulation of 7 genes involved in de novo synthesis of FA (*ACACA*, *FABP3*, *LPL*, *GPAM*, *SREBF1*, *SCD1*, and *FASN*), which is associated with the expected decrease in milk fat secretion [169,170]. These gene expression modifications are related to reduced milk production and milk protein and fat yields. Consistently and more recently, Parreira et al. [159] observed that feed restriction (at a 15–20% reduction in their initial live body weight) for 22 days altered the mammary transcriptomes in 2 breeds of dairy goats with different seasonal weight loss tolerances: Majorera (tolerant) and Palmera (susceptible). In Majorera and Palmera goats, 82 and 99 transcripts were differentially expressed between control and restricted diets, respectively [159]. Feed restriction in both breeds affected several biochemical pathways such as carbohydrate and lipid transport intracellular trafficking, RNA processing, and signal transduction, with higher effects in the Majorera breed. In the Majorera MG, feed restriction leads to decrease in the synthesis of carbohydrates (downregulation of *LALBA* and *GK*, for instance) or lipids (downregulation of *TM7SF2* and *ELOVL6*) in the MG with severe consequences to milk production. These results are a consequence of the reduced metabolic activity of the MG in the restricted group compared to the control group, in accordance with the study of Lérias et al. [157].

The mechanisms underlying the regulation by the nutrition of genes could be explained in part by the nutriregulation of miRNA in the ruminant MG. Indeed, miRNA expression and function have been reported to be modulated by diets involving either deficiency or increased intake [176,177].

The expression of several miRNAs is modified by the stage of lactation. The expression of 12 miRNAs (*miR-10a*, *miR-15b*, *miR-16*, *miR-21*, *miR-33b*, *miR-145*, *miR-146b*, *miR-155*, *miR-181a*, *miR-miR-205*, *miR-221*, and *miR-233*) in bovine MG was greater during negative energy balance (i.e., early lactation) than during the positive energy balance period (i.e., dry period) [178]. The upregulation of the expression of *miR-221* and *miR-33b* observed in bovine MG suggests their involvement in the control of cell proliferation or angiogenesis and in lipogenesis in mammary tissue [178]. Indeed, *miR-33b* is a host gene of the *SREBP* gene (known to be a key regulator of lipogenic gene expression) and is involved in its regulation. A similar comparison between early and late lactation miRNAs in goat MG showed 378 differentially expressed miRNAs (whose *miR-181a* and *-299*, *-2483-5P*, and *miR*-200c) [179].

The experimental feed restriction or food deprivation models, which induced negative energy balance, also highlight similar changes in bovine and caprine MG. Feed restriction for 6 days (at 60% of calculated net energy for lactation requirements) modified the expression of 27 miRNAs (with 25 known and 2 predicted) in MG from mid-lactating Holstein cows (Figure 3). Among them, 19 and 8 were down- and upregulated by feed restriction, respectively [174]. Analysis of target genes indicated that the 8 most abundantly expressed miRNAs (*miR-143*, *miR-181a*, *miR-26b*, *miR-200c*, *miR-25*, *miR-200b*, *miR-181b*, and *miR-155*) regulated the expression of genes related to lipid metabolism, mammary remodeling, and stress response, suggesting a potential role of miRNAs in mammary structure and lipid biosynthesis that could explain changes in milk production and composition. In lactating goats (Figure 4), the expression of 30 differentially expressed microRNAs (14 upregulated and 16 downregulated with differentially expressed miRNAs most highly expressed: *miR-126-3p*, *miR-6119-5p*, *miR-let-7c-5p*, *miR-99a-5p*, *miR-125b-3p*, *miR-140-3p*, *miR-409-3p*, *miR-451-5p,* and *miR-660-5p*) was highlighted as modified in MG by 48 h of food deprivation compared to an *ad libitum* diet [151]. The most significantly targeted pathways by the 30 nutriregulated miRNAs are those regulating cellular proliferation and remodeling of MG, which are most likely changed through the action of these miRNAs [151]. Among differentially expressed miRNAs, the increase in miR-99a-5p was predicted to decrease the expression of mTOR, in line with the reduction in mTOR due to a dietary energy restriction diet in mice as described above, thus contributing to the decrease in protein synthesis [96]. *miR-222-3p*, *miR-188-5p*, *miR*-*541-5p,* and *miR-494-3p*, which are differentially expressed after food deprivation, were all predicted to regulate the expression of PTEN, highlighting PTEN as a key actor of the regulation of protein metabolism due to food deprivation [151]. Moreover, several miRNAs regulated by food deprivation have been identified as potentially targeting mRNAs involved in mammary FA metabolism, lipid droplet formation, and/or milk fat globule secretion and thus have a crucial role in the synthesis and secretion of milk components. A comparison of differentially expressed miRNAs obtained during an induced negative energy balance in cows [174] and after 48 h of food deprivation in goats [151] highlighted similarities between these two species and models. These findings suggest that feed restriction and food deprivation might lead to a change in gene expression through the actions of miRNAs linked to cellular growth and proliferation, as well as the remodeling of mammary cells.

The effects of feed restriction on MG were recently extended to proteome studies [158,173,180]. Proteomic analyses showed that a feed restriction (at 48% barley straw and dry matter basis) during 4 days after an intramammary 24-h LPS challenge resulted in 53 differentially expressed proteins in the MG of early lactation Holstein cows [173]. Ten of those genes were upregulated, and 43 were downregulated. Bioinformatics analysis of differentially expressed proteins highlighted the immune process, metabolism (regulation of protein catabolic and carbohydrate metabolic processes) and cell function (such as RNA splicing, translation, or regulation of cell adhesion) altered by feed restriction. The downregulation of proteins such as HNRNPH1, HNRPC, HNRNPA3, PCBP2, YBX1, SNRPA1, and DHX9 involved in the splicing process suggests that splicing is impaired in the MG of restricted cows. Modifications in the abundance of proteins involved in protein folding and post-translational modifications (GANAB, PDIA3, RPN2, RPN1, CCT4, PDIA4, and PPIB), protein catabolic process (PSMD2 and PPP2CA), and carbohydrate metabolism (PAPSS1, RPS27A, and GANAB) were also identified [173]. All these alterations could partially explain the reduced synthesis and secretion of milk protein in restricted cows compared with control cows [164].

Similar data were observed in the MG of two dairy goat breeds differently adapted to seasonal weight loss (the tolerant Majorera and the susceptible Palmera breeds) under control and feed restriction (during 22 d) diets using proteomic analyses [180] or blue-native PAGE and two-dimensional gel electrophoresis [158]. Over 1000 proteins were identified, and 96 showed altered expression either as a result of breed or of feed restriction. Feed restriction decreased the expression of proteins related to protein, carbohydrate, and lipid metabolism in both breeds [158,180]. In the Majorera breed, a decrease in UDP glucose 4 epimerase and cytochrome C oxidase, both involved in glucose metabolism, was observed after feed restriction, which could affect lactose metabolism and hence milk production. The downregulation of *ACLS1* and *FASN* involved in fat biosynthesis was observed in Majorera by feed restriction, in agreement with the decrease in milk fat yields previously shown in these two goat breeds [157]. In Palmera goats, feed restriction downregulated the expression of proteins directly involved in milk production and composition, such as kappa or α casein, α lactalbumin, or in protein secretion. The abundance of protein disulfide isomerase A4 (PDIA4) was also decreased. This protein, involved in protein folding, was also identified in bovines [173]. Other proteins specifically involved in carbohydrate metabolism such as HHIP-like protein 2, ACSS1, or glutamine-fructose 6 phosphate aminotransferase also had decreased expression in the underfed group. However, two proteins related to lipid metabolism (trifunctional enzyme subunit α and aldehyde dehydrogenase) were increased in Palmera goats after feeding restriction. These proteomic data strongly suggest that protein synthesis is impaired by negative energy balance at different levels (translation, folding, and post-translational modifications). The modifications of protein metabolism might partially explain the lower milk protein yield observed during restriction [164]. Thus, natural or induced negative energy balance in ruminants affected multiple aspects of MG function, as demonstrated by modifications of milk secretion and changes in MG gene expression at a transcriptional level in part through the actions of miRNA.

## 9. Conclusions

It is now fully recognized that among environmental factors that play a significant role in MG development and subsequent lactation, nutrition and, more specifically, diet composition are of utmost importance. Indeed, changes in energy intake and/or diet composition affect multiple aspects of MG function, as demonstrated by modifications of milk secretion and composition, changes in MG gene expression at a transcriptional level in part linked to cellular growth and proliferation, as well as the remodeling of mammary cells and alterations of MG protein expression. The variations in feeding components and challenges as well as the techniques used to assess diet-induced outcomes undoubtedly contribute to understanding the variety of phenotypes reported in this review. Knowledge from three species, frequently used as models for mammary biology investigations, clearly demonstrates the importance of critical physiological stages, such as puberty, gestation, and early lactation, in the nutritional regulation of mammary development, insofar as it is tightly dependent on hormonal cross-talks. Similarly, studies on lactating model animals as well as on dairy livestock animals, regarding their similarities or differences in response to nutritional challenges, allow to assess not only the total energetic value of the diet but also the relative importance of the various dietary nutrients, in particular their dose-dependent effects as well as their interactions in the ration.

Nevertheless, the elucidation of the fundamental underlying mechanisms at the gene and cellular levels remains incomplete; in particular, the influence of miRNA and epigenetic modulation must be clearly specified. Moreover, up to now, most of the research aiming to decipher the nutritional regulation of milk components synthesis and secretion was carried out on mammary tissue, which is a complex tissue constituted of several cellular types. However, the influence of each type of cells is significant and specific. For example, a recent study suggested that the expression of miRNAs could be specific to different cells [181]. The access to single cell RNA-seq, already used in breast cancer studies [182,183], will provide a deeper understanding of fine regulatory mechanisms of milk synthesis.

Accumulating evidence in both humans and animals demonstrates that nutritional influences encountered during early life have a lasting impact on both health and performance, including milk quantity and quality. From this perspective, by transmitting bioactive factors from mother to offspring, milk may play a key role in the programming process, helping to ensure healthy developmental outcomes in offspring [184] and providing good footprints to prepare healthy adults. A clear challenge is therefore required to define the mechanistic dynamics involved and their regulation by a multidisciplinary and systems biology approach, considering molecular, cellular, physiological, and environmental dimensions. Furthermore, the understanding of diet-induced phenotypes may open the door to opportunities for interventions to improve both animal health and performance potential as well as the health of milk consumers.

## Abbreviations

(CLA)	conjugated linoleic acid
(DHA)	docosahexaenoic acid
(FA)	fatty acid
(MG)	Mammary Gland
(miRNA)	microRNA
(RNA-seq)	RNA sequencing
